# Reactive and Proactive Aggression among Children and Adolescents: A Latent Profile Analysis and Latent Transition Analysis

**DOI:** 10.3390/children9111733

**Published:** 2022-11-11

**Authors:** Annette Lohbeck

**Affiliations:** Institute for Educational Quality Improvement (IQB), Humboldt-University of Berlin, 10117 Berlin, Germany; annette.lohbeck@iqb.hu-berlin.de

**Keywords:** reactive and proactive aggression, profiles, latent profile analysis, latent transition analysis

## Abstract

The present study aimed to explore children’s and adolescents’ profiles of reactive and proactive aggression and the stability of those profiles over a six-month period using latent profile analysis (LPA) and latent transition analysis (LTA). Data were collected at two measurement points from a sample of *N* = 1468 children and adolescents aged from 9 to 18 years. Results of LPA revealed three distinct profiles, labeled as “Severe Reactively and Proactively Aggressive (S-RA-PA)”, “Highly Reactively and Proactively Aggressive” (H-RA-PA), and “Moderately Low Reactively and Proactively Aggressive” (M-RA-PA). All profiles appeared to be relatively stable over six months, supporting their within-sample consistency. The most stable and largest profile was the “M-RA-PA” profile, while the least stable and smallest profile was the “S-RA-PA” profile. However, there was also some within-person variability in children’s and adolescents’ profile membership because almost 40–50% of the participants of the “S-RA-PA” and “H-RA-PA” profiles transitioned to another profile across six months. In contrast, more than 90% of the participants of the “M-RA-PA” profile remained in their profile. These results provide a dynamic picture of children’s and adolescents’ development of reactive and proactive aggression and bear several implications from a short longitudinal person-oriented perspective.

## 1. Introduction

The differentiation between various types of aggression is of considerable importance not only for describing and explaining aggressive behavior but also for predicting psychological characteristics and planning interventions. A key differentiation of aggressive behavior, which has become increasingly important in the last two decades, is between reactive and proactive aggression [1,2,3,4,5]; see for a review [6]. Reactive aggression is conceptually based on the frustration-aggression hypothesis [7,8] and describes a retaliatory defensive response to a perceived threat, provocation, or frustration accompanied by feelings of anger [1,9]. In contrast, proactive aggression is based on the concept of social learning theory [10,11] and represents a non-provoked, intentional, instrumental, and offensive behavior that is acquired or maintained through reinforcement processes [1,9]. However, most studies dealing with reactive and proactive aggression of children and adolescents have been variable-oriented, exploring the average relations between variables in a specific sample and neglecting the combined effects of various types of aggression at the individual level. In contrast, person-centered approaches such as latent profile analyses (LPA) can systematically test for the combined effects of different types of aggression because they seek to identify subpopulations (or profiles) characterized by distinct configurations on a set of interacting variables [12,13]. Despite the increased application of LPA, person-oriented studies on reactive and proactive aggression have been scarce, and very little research has yet tested the stability of latent profiles of various subtypes of reactive and proactive aggression among children and adolescents. For this reason, the present study was specifically designed to (1) identify children’s and adolescents’ profiles of reactive and proactive aggression using LPA and (2) test for the stability of those profiles among children and adolescents using latent transition analysis (LTA). The incremental contribution of this research is, in particular, that four rather neglected subtypes of reactive and proactive aggression were considered as profile indicators [14,15], namely: (1) anger-aggression (i.e., aggressive behavior due to anger feelings) and (2) defensive aggression attribution (i.e., aggressive behavior combined with a defensive attribution of aggression) for reactive aggression, and (3) resource acquisition (i.e., aggressive behavior for the purpose to covet to appropriate resources) as well as (4) exercise of power/dominance for proactive aggression (see also [16,17]). Until now, no person-oriented research has yet examined children’s and adolescents’ profiles and their stability based on these four subtypes of reactive and proactive aggression in one empirical study simultaneously. Thus, the present study expands upon previous research and enriches interpretations of the heterogeneity in children’s and adolescents’ reactive and proactive aggression. Finally, this study also bears several implications for future research and interventions aiming at reducing reactive and proactive aggression among children and adolescents, which may play an important role in children’s and adolescents’ healthy mental development.

## 2. Reactive and Proactive Aggression of Children and Adolescents

Numerous studies have shown that reactively and proactively aggressive children exhibit different deficits in social information processing [4,9,18,19,20]: While reactively aggressive children have weaknesses in encoding and interpreting social information [18,21] and are more likely to ascribe hostile intentions in ambiguous situations [1,9,22], proactively aggressive children pursue instrumental rather than relational goals, evaluate aggression more positively [9], and expect positive outcomes after aggressive behavior [9,21,22]. Furthermore, reactive aggression has frequently been found to be closely related to increased sensitivity to stress stimuli [23], attention problems [24,25], impulsivity [26], social rejection by peers [21,27,28,29], and peer victimization [22,30]. Reactively aggressive children are more likely to exhibit internalized problems that are caused by a lack of regulation of emotions, especially anger (cf., [31,32]; see for a review also [33,34]). In contrast, proactively aggressive children often show assertiveness [22], leadership [30], and a sense of humor [1]. For instance, Dodge et al. [21] found higher self-efficacy beliefs in social situations and greater self-esteem in proactively aggressive children than in reactively aggressive children. Finally, proactive aggression is also highly related to externalized problems, such as adolescent delinquency and alcohol abuse ([35]; see for an overview, [36]). These results indicate that reactive and proactive aggression are differentially linked to various outcomes.

## 3. Aggression Profiles and Their Stability

Research exploring aggression profiles in childhood and adolescence has largely been neglected (e.g., [37,38,39]). Most of the available research has used cluster analysis (e.g., [40,41,42]) investigating reactive and proactive aggression more generally. For instance, using cluster analysis, Euler, Steinlin, and Stadler [43] identified three clusters of reactive and proactive aggression in a sample of *N* = 254 adolescents aged from 12 to 18 years: (1) a “reactive-proactive aggression” cluster with elevated scores on reactive aggression, proactive aggression and total aggression, (2) a “reactive aggression” cluster with higher scores on reactive aggression only, and (3) a “low aggression” cluster with the lowest scores on all three types of aggression. These results are also in line with results by Crapanzano, Frick, and Terranova [44], who observed no proactive-only group but several severity-based subgroups of aggression among *N* = 282 children and adolescents aged 9 to 14 years. More specifically, based on cluster analyses, results revealed (1) an aggressive cluster with low levels of reactive aggression and (2) an aggressive cluster with high levels of both reactive and proactive aggression.

Rather inconsistent findings can also be found in person-oriented research based on LPA (e.g., [45,46,47]). For instance, Smeets et al. [46] examined latent profiles of reactive and proactive aggression among 587 adolescents aged from12 to 20 years and identified four aggression profiles based on three indicators of reactive and proactive aggression (i.e., proactive aggression, reactive aggression due to internal frustration, and reactive aggression due to external provocation): (1) a profile with low levels of all three types of aggression (*n* = 220; 37.5%), (2) a “moderate reactive aggression” profile with moderate levels of both types of reactive aggression and low levels of proactive aggression (*n* = 222; 37.8%), (3) a “proactive and reactive aggression” profile with higher levels of both types of reactive aggression and average levels of proactive aggression (*n* = 97; 16.5%), and (4) a “severe proactive and reactive aggression” profile with higher levels of all three types of aggression (*n* = 47; 8%). Intriguingly, all profiles had significantly lower levels of proactive aggression than both reactive aggression factors, and there was no proactive-only profile with only high levels of proactive aggression.

In contrast, Kokkinos et al. [45] found three distinct profiles in a non-clinical sample of 301 Greek early adolescents: Profile 1 (*n* = 161, 54%), labeled as “low aggression”, was characterized by low levels of both reactive and proactive aggression. Profile 2 (*n =* 98, 33%), labeled as “high reactive aggression”, was dominated by high levels of reactive aggression and moderate levels of proactive aggression, whereas Profile 3 (*n* = 41, 13%), labeled as “combined”, was characterized by high levels of both reactive and proactive aggression. However, all these studies were only cross-sectional, and there are still no studies testing the stability of children’s and adolescents’ aggression profiles based on various subtypes of reactive and proactive aggression. In contrast, numerous studies have investigated the stability of latent profiles using total scores on aggression or different aggression indicators. For instance, drawing on three indicators of aggression (i.e., social rejection, affiliation with aggressive peers, and academic failure), Jung, Krahé, and Buschmann [48] identified three distinct profiles in a sample of *N* = 1479 children and adolescents aged 9–19 years: a non-risk group and two risk groups, labeled as the social rejection group and the affiliation with aggressive peers/academic failure group. Results of a latent path analysis revealed relatively high stability of profile membership of the risk groups. Similar findings were also reported by Evans, Dίaz, Callahan, Wolock, and Fite [49], who considered the trajectories of reactive and proactive aggression in a sample of 1420 children aged 5–12 years from grades 3–5 over six school years. Latent class growth models yielded four trajectory profiles: Profile 1, named “Low Aggression” (76.7% of the sample), showed a rather normative pattern, with decreasing levels of proactive aggression and consistently low levels of reactive aggression over time. In contrast, Profile 2 (4.7% of the sample), labeled as “High Proactive-Reactive Aggression,” was characterized by highly stable reactive aggression and highly increasing proactive aggression but also by a high seasonal effect for both aggression types, with higher levels of aggression in the spring period than in the autumn period. Profile 3 (4.9% of the sample), named “Declining Aggression”, exhibited the highest levels of both types of aggression, which decreased over time, whereas Profile 4 (13.7% of the participants), labeled as “Predominantly Reactive Aggression,” was characterized by highly stable levels of reactive aggression over time, accompanied by slightly increasing low levels of proactive aggression over time. These and many other findings provided support for the relatively high stability of children’s and/or adolescents’ profiles of reactive and proactive aggression (e.g., [50,51,52,53]).

To resume, although previous research exploring children’s and adolescents’ profiles of reactive and proactive aggression has been rather inconsistent, three aggression profiles have most frequently been observed in the literature, i.e., profiles with either high, moderate, or low levels of both reactive and proactive aggression. In contrast, research investigating the stability of children’s and adolescents’ profiles of reactive and proactive aggression seems to be more consistent, indicating relatively high stability of latent aggression profiles among children and adolescents. However, none of the available studies have yet considered different subtypes of reactive and proactive aggression as profile indicators in LPA and LTA.

## 4. Aims and Hypotheses

Using LPA and LTA, the present short-time longitudinal study is unique in examining children’s and adolescents’ profiles of reactive and proactive aggression based on four subtypes of reactive and proactive aggression (i.e., anger-aggression, defensive aggression attribution, resource acquisition, exercise of power/dominance). In sum, the following two central research questions were tested in this study:

- Research question 1: Which profiles of reactive and proactive aggression based on four subtypes of reactive and proactive aggression (i.e., anger-aggression and defensive aggression attribution for reactive aggression; resource acquisition and exercise of power/dominance for proactive aggression) can be found among children and adolescents?
- Research question 2: Are children’s and adolescents’ profiles of reactive and proactive aggression stable over time (i.e., six months)?

Despite the great inconsistency of previous research, three aggression profiles were expected, which have most frequently been found among children’s and adolescents’ aggression in the literature: a high-aggression profile, a moderate-aggression profile, and a low-aggression profile (Hypothesis 1). Furthermore, in support of some earlier research (e.g., [50,51,52,53]), all three profiles were assumed to be relatively stable over six months (Hypothesis 2). Finally, above and beyond the two central objectives and hypotheses, the present study also attempted to explore the possible sex and age differences in children’s and adolescents’ profiles of reactive and proactive aggression. Since several studies have provided evidence for systematic sex differences in reactive and proactive aggression in favor of girls (e.g., [2,40,54]), boys were assumed to show more unfavorable aggression profiles than girls (Hypothesis 3). In contrast, due to inconsistency and/or absence of solid research, no specific hypotheses concerning significant age differences were posited.

## 5. Methods

### 5.1. Sample

A sample of *N* = 1468 children and adolescents (*n* = 764 boys and *n* = 704 girls) aged from 9 to 18 years (*M* = 13.11, *SD* = 2.33; age 9: *n* = 94; age 10: *n* = 165; age 11: *n* = 151; age 12: *n* = 186; age 13: *n* = 195; age 14: *n* = 204; age 15: *n* = 230; age 16: *n* = 150; age 17: *n* = 67; age 18: *n* = 26) participated at the first measurement point in this study. In contrast, the sample of the second measurement point included *N* = 208 children and adolescents (boys: *n* = 103; girls: *n* = 105) aged from 9 to 17 years (*M* = 12.35, *SD* = 2.40; age 9: *n* = 41; age 10: *n* = 17; age 11: *n* = 21; age 12: *n* = 30; age 13: *n* = 23; age 14: *n* = 23; age 15: *n* = 34; age 16: *n* = 15; age 17: *n* = 4). The large participant attrition resulted from the piling up of events in many schools (e.g., report card conferences and class trips) and the fact that several test administrators were no longer available at the second measurement point. All participants were recruited from different elementary and secondary schools in Germany (i.e., nine schools at T1: four elementary schools: *n* = 150, 10.2%, three mixed school tracks: *n* = 797, 54.3%, and two highest academic school tracks: *n* = 521, 35.5%; four schools at T2: one elementary school: *n* = 83, 39.9%, three mixed school tracks: *n* = 125, 60.1%). All schools were randomly selected in four federal states of Germany. More precisely, the schools participating in T1 were located in Bremen, Lower Saxony, North Rhine-Westphalia, and Saxony, while the schools participating in T2 were all situated in Lower Saxony.

### 5.2. Procedure

Data collection took place as part of a standardization study of another questionnaire for measuring students’ social and learning behavior at the beginning of February 2014 (T1) and the end of July 2014 (T2). First, the first author contacted the schools via email and phone. Second, the dates for the data collection were fixed with the school principals and involved class teachers, and a letter to the parents of the participating students was distributed one week before the data collection. Participation was voluntary and anonymous. Written informed consent was obtained from the school principals, the class teachers, and the parents of the children. At both measurement points, all children and adolescents filled out the questionnaires in one regular lesson at school (i.e., 45 min). Undergraduate research assistants administered the testing in class and advised the students that their answers would be treated confidentially. Due to the great variety in younger children’s reading abilities, all items were read aloud in class by a trained test leader to avoid interfering effects on reading ability. Data collection was approved by the Ministries of Education in the relevant countries and the school principals of the schools where the data were collected. All procedures were performed according to the ethical principles and human subjects’ guidelines of the American Psychological Association [55].

### 5.3. Measures

Reactive/proactive aggressive behavior. The four subtypes of reactive and proactive aggression were measured with the four scales of the “Differential Aggression Questionnaire” (in German: “Differentieller Aggressionsfragebogen; DAF, [56]). This questionnaire is a sufficiently tested, standardized self-report measure for children and adolescents, which comprises sixteen items, with four items forming one scale and two scales measuring reactive and proactive aggression, respectively. For reactive aggression, the two scales “anger-aggression” (e.g., How often have you thrown a tantrum? T1/T2: Cronbach *α* = 0.74/0.77) and “defensive aggression attribution” (e.g., How often have you fought even though you did not want to? T1/T2: Cronbach *α* = 0.74/0.76) were used, while for proactive aggression, the two scales “resource acquisition” (e.g., How often have you threatened someone to do what you want? T1/T2: Cronbach *α* = 0.80/0.85) and “exercise of power/dominance” (e.g., How often have you hit someone just for fun? T1/T2: Cronbach *α* = 0.67/0.79) were applied (see also the items and scales in the Supplementary Materials). All items were formulated as questions and responded to on a 4-point response scale ranging from “never” (0), “rarely” (1), “more often” (2), to “often” (3). Support for the reliability and validity of this self-report measure can be found in various studies (e.g., [14,15,57]). Furthermore, results of confirmatory factor analysis (CFA) also provided strong support for the superiority of a 4-factor CFA model as opposed to a 1-factor CFA model including one factor for all four subtypes of aggression and a 2-factor CFA model positing one factor for both subtypes of reactive aggression and a second factor for both subtypes of proactive aggression (see Table 1).

### 5.4. Data Analyses

All analyses were performed in M*plus* 8.7 [58] using the robust maximum likelihood estimator (MLR). When testing the first research question asking for the number of profiles among children and adolescents, one to six LPA solutions were estimated based on the four aggression factors as profile indicators for each measurement point separately. Means and variances for these indicators were freely estimated across the profiles. To provide some control for measurement errors, all LPA models were tested with factor scores (*M* = 0, *SD* = 1) and a number of 5000 random sets of start values, 1000 iterations, and the 200 best solutions for the final stage optimization [12]. Missing data were handled with the full information maximum likelihood approach (FIML). The number of missing values varied from 6.4 to 19.1%. The selection of the optimal number of profiles was based on the meaning, the theoretical conformity, and the statistical adequacy of the solutions, as well as multiple statistical indicators [12], i.e., the Akaike Information Criterion (AIC), the Bayesian Information Criterion (BIC), the Consistent AIC (CAIC), the Sample-Size-Adjusted BIC (SSABIC), the adjusted Lo-Mendell-Rubin (aLMR) likelihood ratio test, and the Bootstrap Likelihood Ratio Test (BLRT). A better fitting profile solution was assumed if it showed lower values on the AIC, BIC, CAIC, and SSABIC than the profile solution with one less profile. Furthermore, the *p*-values of the aLMR and BLRT, as well as entropy (i.e., classification accuracy), were also considered. The superiority of a model including one less profile was supported by a non-significant *p*-value of the aLMR and BLRT, with a high value of the entropy, ranging from 0 (low) to 1 (high). When the best LPA solution was selected, the second research question asking whether children’s and adolescents’ profiles would be stable over six months, was examined. For this purpose, the final LPA solution was converted to an LTA model testing the within-person stability of the profiles (i.e., whether children and adolescents correspond to the same profile over the two measurement points). More precisely, to determine the within-sample stability of the profiles (or longitudinal profile similarity across time), an LTA model with measurement non-invariance (i.e., assuming configural similarity) was compared to an LTA model with full measurement invariance (i.e., constraining all parameters to be equal over six months; [59]). Measurement invariance (or profile stability) was established if the more restricted model, as opposed to the less restricted model, showed lower values of at least two indices out of the CAIC, BIC, and SSABIC [12]. Finally, to reveal the possible sex and age differences in children’s and adolescents’ profiles of reactive and proactive aggression, a multiple multinomial logistic regression model was tested, with sex and age as auxiliary variables, using the auxiliary option in M*plus* [12].

## 6. Results

### 6.1. LPA

Results of LPA including one to six latent profiles estimated separately for each measurement point are presented in Table 2. At both measurement points, the AIC, ABIC, and BLRT kept on improving when adding further profiles to the data. While the *p*VMRT indicated a significantly better fit of the 2- and 5-profile solutions compared to the 1- and 4-profile solutions, the *p*BLRT suggested a significantly better fit of all profile solutions (i.e., up to the 6-profile solution). However, when adding a third profile to the data, another well-defined qualitatively distinct and theoretically meaningful profile appeared, which was not observed in the 2-profile solution and characterized by very high levels of proactive aggression, accompanied by relatively high levels of reactive aggression. In contrast, adding a fourth profile to the data resulted in the arbitrary division of one of the existing profiles into two distinct profiles differing only quantitatively from one another. For this reason, the 3-profile solution was finally selected for further analyses, which also provides a reasonable level of classification accuracy with an entropy value ranging from 0.955 (T1) to 0.981 (T2).

**Figure 1:**
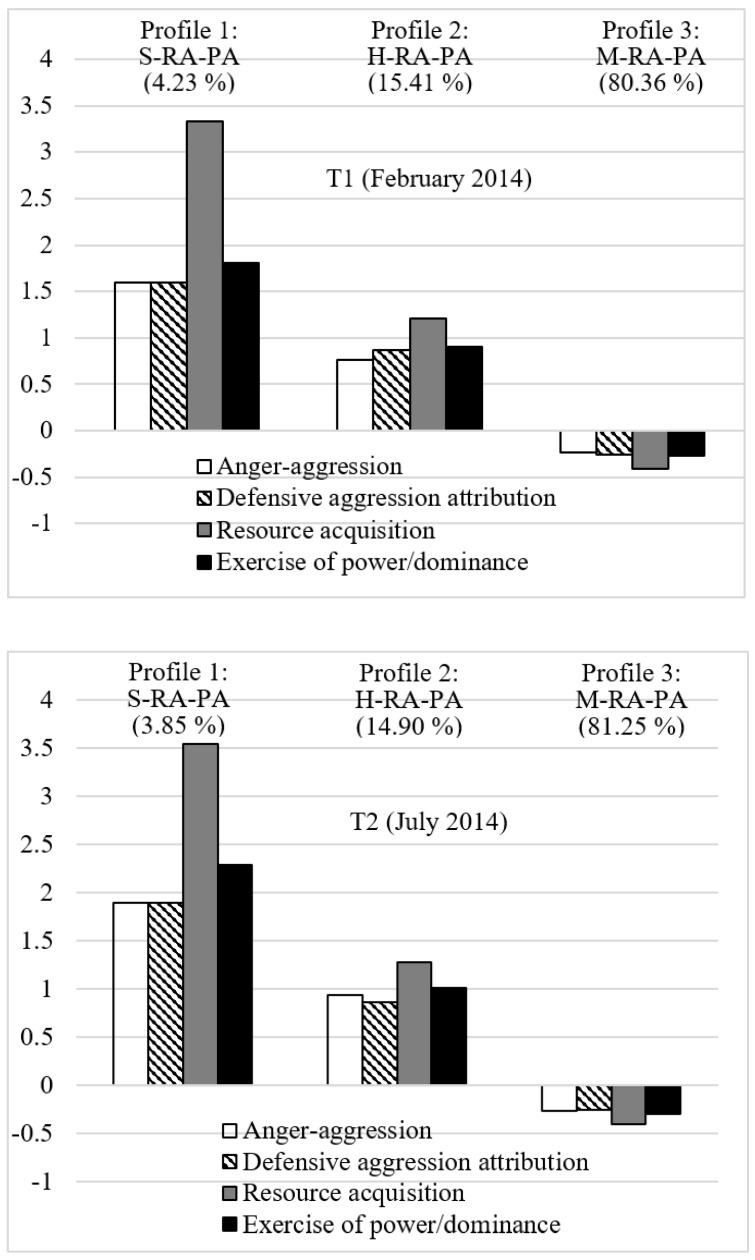
Final 3-profile solution for both time points. *Note*. The profile indicators are estimated from factor scores with a mean of 0 and a standard deviation of 1. S-RA-PA = “Severe Reactively and Proactively Aggressive” profile, H-RA-PA = “Highly Reactively and Proactively Aggressive” profile, M-RA-PA = “Moderately Low Reactively and Proactively Aggressive” profile.

When characterizing the three profiles, both the mean levels and severity (or intensity) of the four subtypes of reactive and proactive aggression were considered. Profile 1, labeled as “Severe Reactively and Proactively Aggressive (S-RA-PA)”, included 4.23% or 3.85% (T1/T2: *n* = 62/8) of the participants. This profile was dominated by very high levels of both proactive aggression factors, accompanied by high levels of both reactive aggression factors. Profile 2, called “Highly Reactively and Proactively Aggressive (H-RA-PA)”, involved 15.41% or 14.90% (T1/T2: *n* = 226/31) of the sample. This profile showed relatively high levels of all four aggression indicators, with the highest levels of resource acquisition. Finally, Profile 3, labeled as “Moderately Low Reactively and Proactively Aggressive (M-RA-PA)”, comprised 80.36% or 81.25% (*n* = 1179/169) of the participants. This profile exhibited relatively moderate levels of all four aggression indicators. Intriguingly, all these three profiles did not differ in the four subtypes of reactive and proactive aggression but in terms of the severity of each subtype of those two aggression factors. In detail, children and adolescents in the first two more unfavorable profiles 1 and 2 (i.e., S-RA-PA and H-RA-PA) demonstrated the highest level of proactive aggression, i.e., resource acquisition, followed by the exercise of power/dominance, while they showed rather equal levels of anger-aggression and defensive aggression attribution within their profile. In contrast, children and adolescents of the more favorable “Moderately Low Reactively and Proactively Aggressive (M-RA-PA)” profile 3 presented the lowest level of proactive aggression, i.e., resource acquisition, followed by the exercise of power/dominance, defensive aggression attribution, and anger-aggression within their profile. Table 3 and Figure 1 provide the standardized mean levels of the four aggression factors for all three profiles under investigation.

### 6.2. LTA

When exploring the transition probabilities to the T2 profiles, the LPA 3-profile solution was converted to an LTA model. The results of this model are presented in Table 4. Both the “Severe Reactively and Proactively Aggressive (S-RA-PA)” profile (Profile 1) and “Highly Reactively and Proactively Aggressive (H-RA-PA)” profile (Profile 2) showed a rather moderate level of within-person stability in children’s and adolescents’ profile membership because almost 40-50% of the participants of these two profiles remained in their profile at T2. In contrast, the “Moderately Low Reactively and Proactively Aggressive (M-RA-PA)” profile (Profile 3) demonstrated the highest stability rate. In detail, the “Severe Reactively and Proactively Aggressive (S-RA-PA)” profile (Profile 1) only transitioned to the “Highly Reactively and Proactively Aggressive (H-RA-PA)” profile (i.e., Profile 2; 54.7%). In contrast, the “Highly Reactively and Proactively Aggressive (H-RA-PA)” profile (Profile 2) mainly migrated to the “Moderately Low Reactively and Proactively Aggressive (ML-RA-PA)” profile (i.e., Profile 3; 37.5%) and less to the “Severe Reactively and Proactively Aggressive (S-RA-PA)” profile (i.e., Profile 1; 4.1%). Finally, the “Moderately Low Reactively and Proactively Aggressive (M-RA-PA)” profile (Profile 3) mainly moved to the “Highly Reactively and Proactively Aggressive (H-RA-PA)” profile (i.e., Profile 2; 6.6%), whereas only a low proportion of the participants transitioned to the “Severe Reactively and Proactively Aggressive (S-RA-PA)” profile (Profile 1; 0.6%). However, results of measurement invariance testing provided strong support for the full measurement invariance across time of the LTA solution and thus high profile stability, as all fit indicators (i.e., CAIC, BIC, and SSABIC) of the LTA model assuming full measurement invariance were lower than those of the LTA model without full measurement invariance. These results indicated that all measurement parameters were equal over six months.

### 6.3. Sex and Age Differences in the Three Aggression Profiles

When testing the possible sex and age differences in the three aggression profiles, results of the multiple multinomial logistic regression model revealed some significant sex differences in the three aggression profiles at T1 but non-significant sex and age differences at both measurement points. Table 5 provides the results of this analysis.

At T1, boys were more likely to be members of the most unfavorable “Severe Reactively and Proactively Aggressive (S-RA-PA)” profile (Profile 1) relative to the “Moderately Low Reactively and Proactively Aggressive (M-RA-PA)” profile (Profile 3) than girls. Furthermore, boys had a greater likelihood of membership in the “Highly Reactively and Proactively Aggressive (H-RA-PA)” profile (Profile 2) than in the “Moderately Low Reactively and Proactively Aggressive (M-RA-PA)” profile (Profile 3) when compared to girls. In contrast, there were non-significant sex and age differences in the three profiles at T2.

## 7. Discussion

The present study expanded upon previous research by (1) exploring children’s and adolescents’ profiles of reactive and proactive aggression using LPA based on four subtypes of reactive and proactive aggression (i.e., anger-aggression and defensive aggression attribution for reactive aggression; resource acquisition and exercise of power/dominance for proactive aggression), (2) investigating possible sex and age differences in those profiles, and (3) testing the short-time stability of those profiles over six months using LTA.

Results of LPA revealed three qualitatively distinct and theoretically meaningful profiles, labeled as “Severe Reactively and Proactively Aggressive (S-RA-PA)” profile (Profile 1), “Highly Reactively and Proactively Aggressive (H-RA-PA)” profile (Profile 2), and “Moderately Low Reactively and Proactively Aggressive (M-RA-PA)” profile (Profile 3). In contrast, there were no reactive-only or proactive-only aggression profiles, dominated by very high levels of reactive or proactive aggression, as also earlier reported in some research (e.g., [44,46]). These results provide strong support for the co-existence of reactive and proactive aggression, as also stated in numerous studies (for a meta-analysis, see [5]). Thus, if children and adolescents perceive high levels of reactive aggression, they are also more likely to perceive high levels of proactive aggression. This is an important finding for further research and intervention programs with aggressive children or adolescents because it suggests that reducing reactive aggression may also lower proactive aggression or vice versa. However, results also demonstrated that the levels of both subtypes of proactive aggression were more pronounced than those of both subtypes of reactive aggression in the two more unfavorable profiles 1 and 2, while those of both subtypes of reactive aggression were rather similar in all three profiles. The highest level was found for resource acquisition in these two unfavorable profiles. It seems that children and adolescents perceive proactive aggression more frequently than reactive aggression and focus on gaining more resources or advantages when harming their peers. However, these are very worrying findings because higher levels of proactive aggression are more frequently related to more severe forms of aggression or psychopathic personality development [26]. As a consequence, intervention programs should primarily aim at reducing children’s and adolescents’ proactive aggression and resource acquisition. The three aggression profiles in this study also align well with those reported in other previous studies (e.g., [43,46,60]) showing at least three aggression profiles with low, moderate, or high levels of both reactive and proactive aggression. However, the most striking but also alarming finding of this study was that there were two rather unfavorable aggression profiles characterized by (very) high levels of both reactive and proactive aggression. Although most of the participants were members of the more favorable “Moderately Reactively and Proactively Aggressive (M-RA-PA)” profile (>80%), there was also a substantial number of participants (i.e., 15–20%) with one of the two rather unfavorable profiles. This result must also be considered very disturbing because higher levels of reactive and proactive aggression can lead to serious and pervasive problems in later childhood and adolescence [20], such as adolescent alcohol use, delinquency, and psychopathy ([61]; see for a meta-analysis, [62] or [33], and for an overview, [36]).

Intriguingly, the number and structure of the three profiles appeared to be quite similar between the two measurement points in this study, providing support for their within-sample stability. More specifically, the LTA model assuming full measurement invariance of the three profiles showed lower fit indices than the LTA model positing non-invariance of the parameters across time. These findings are also consistent with Hypothesis 2 and other previous results (e.g., [48,50,51,52,53]) stating relatively high stability of children’s and adolescents’ reactive and proactive aggression profiles across time. This research is of considerable importance for intervention programs targeting reducing children’s and adolescents’ reactive and proactive aggression because when profiles vary across time, intervention strategies may not be very effective. In contrast, stable profiles could have a substantial and persistent impact on children’s and adolescents’ development.

Even more reassuring were the results of the LTA in this study, providing evidence for some variability of the within-person stability of the two rather unfavorable aggression profiles (i.e., S-RA-PA, M-RA-PA): almost 40–50% of the participants of the two profiles mainly moved to more socially acceptable responses at T2 within six months. More precisely, the “Severe Reactively and Proactively Aggressive (S-RA-PA)” profile (Profile 1) mainly transitioned to the “Highly Reactively and Proactively Aggressive (H-RA-PA)” profile (Profile 2) at T2, whereas the “Highly Reactively and Proactively Aggressive (H-RA-PA)” profile (Profile 2) mainly moved to the “Moderately Reactively and Proactively Aggressive (M-RA-PA)” profile (Profile 3) at T2. This result also confirms the research by Evans et al. [49] highlighting that the level of aggression in children in the spring period was usually higher than in the autumn period. In contrast, the most desirable profile was the most stable in this study because more than 90% of the participants remained in this profile at T2. However, the “Severe Reactively and Proactively Aggressive (S-RA-PA)” profile (Profile 1) did not show a transition to this more favorable profile at all. By implication, children and adolescents of the “Severe Reactively and Proactively Aggressive (S-RA-PA)” profile (Profile 1) are at risk and need to be supported in intervention programs.

When additionally exploring the possible sex and age differences in children’s and adolescents’ profiles of reactive and proactive aggression, there was strong support for some significant sex differences in the three profiles at T1 but no support for significant sex and age differences at both measurement points. In line with Hypothesis 3, boys were more likely to be members of the two more unfavorable “Severe Reactively and Proactively Aggressive (S-RA-PA)” profile (Profile 1) and “Highly Reactively and Proactively Aggressive (H-RA-PA)” profile (Profile 2) than girls. This result also corresponds to previous research showing generally higher levels of reactive and proactive aggression among boys (e.g., [2,40,54]). In contrast, the non-significant age differences at both measurement points suggest that children’s and adolescents’ profiles of reactive and proactive aggression do not vary across age, thus, providing evidence of a high consistency of those two types of aggression across age, as also reflected by the results of LTA estimated in this study. A possible reason for the non-significant age differences may be the great age range in this study because all ages from 9 to 18 years were included in the sample. However, due to this great age range, the number of participants was very low in each age group (*n* < 200), not allowing for comparisons between specific age groups. For this reason, further research must show whether children’s and adolescents’ profiles of reactive and proactive aggression may also differ across specific age groups.

### 7.1. Limitations and Future Directions

Several limitations must be noted. First, the sample of this study was focused on a sample of students from Germany, which was not representative of the whole country, as only three regions at T1 and a single region at T2 were taken into account, limiting the generalizability of the results. Second, there was significant participant attrition at T2, which reduced the overall number of participants from 1468 to 208. Thus, the reported findings on the stability of the three profiles must be considered rather tentative and speculative in this study. However, although the T2 profiles were small (i.e., *n* = 8 for profile 1, *n* = 31 for profile 2, and *n* = 169 for profile 3) and the period between the two measurement points was very short (i.e., six months), an interesting finding of this research was that there were three qualitatively and quantitatively distinct profiles with rather equal levels of all four subtypes of proactive and reactive aggression at both measurement points. Third, apart from some demographic data, only one short tool for measuring proactive and reactive aggression was used, which does not allow for drawing conclusions in social sciences. In addition, both aggression factors were only measured through children’s and adolescents’ self-reports, which may be more affected by self-evaluation bias than positive characteristics. Future studies should also include further measures or data of, for instance, teachers, parents, and peers to validate children’s and adolescents’ self-evaluations. Finally, the particular focus of this study was on the identification of children’s and adolescents’ profiles of reactive and proactive aggression and the stability of those profiles over six months, but not on the potential predictors and outcomes of these profiles. An interesting research question is, in particular, which familial factors play the most important role in the prediction of the four subtypes of reactive and proactive aggression, such as parental assistance with emotion regulation in early childhood or parental encouragement of aggressive behavior in adolescence [20,61,63]. In summary, further research is needed to reveal the possible precursors and outcomes of children’s and adolescents’ profiles based on the four subtypes of reactive and proactive aggression using more heterogenous samples and considering a larger time period or more than two measurement points.

### 7.2. Implications and Conclusions

Despite the limitations mentioned, this study makes an incremental contribution to previous literature by showing three qualitatively distinct and relatively stable profiles of children’s and adolescents’ reactive and proactive aggression based on four subtypes of these two aggression factors. However, the most important but also alarming finding of this study was that there were two rather unfavorable aggression profiles characterized by (very) high levels of both subtypes of proactive aggression. This research implies that a substantial number of children and adolescents are at risk and need to be supported in intervention programs, even though this profile appeared to be the least stable in this study. An effective approach for interventions with reactively and proactively aggressive children and adolescents is, for instance, to enhance children’s and adolescents’ social and emotional skills (e.g., empathy) as well as emotional regulation [64]. However, since reactive and proactive aggression are clearly empirically separable from each other and have been found to be differentially linked to various precursors and outcomes (e.g., [20,26,65,66]; see for a review, [6]), specific treatments and intervention programs should be tailored for reactively and proactively aggressive children and adolescents (cf., [66]). For instance, some beneficial strategies to reduce reactive aggression are the modification of hostile attributions, the desensitization to threats, and social skills training, in which reactively aggressive children and adolescents can learn to interpret social information adequately. Furthermore, since reactive aggression can also result from inhibition problems, the application of pharmacological strategies may also reduce children’s and adolescents’ reactive aggression. In contrast, as proactively aggressive children and adolescents are more likely to use operant tactics [67], these children and adolescents may particularly benefit from skills training, in which they learn to link positive consequences after non-aggressive behavior, such as feeling accepted by peers [68].

To conclude, the results of this study suggest important perspectives on children’s and adolescents’ proactive and reactive aggression based on a self-report measure, even though the results on the stability of the three profiles have still to be supported by further longitudinal studies. However, in summary, the reported findings highlight the co-existence of three profiles of proactive and reactive aggression: the first profile with more proactive and less reactive children and adolescents, the second profile with both reactive and proactive children and adolescents, and the third profile including children and adolescents with both less reactive and proactive aggression levels. An important finding of this research was, in particular, that resource acquisition achieved the most pronounced level in all three profiles, which prompts to undertake interventions in this subtype of proactive aggression. Thus, the likelihood of reactive and proactive aggression co-existing may be an important perspective for further understanding and for guiding appropriate support and prevention through early intervention, even though the distinction between reactive and proactive aggression may also be of great significance when establishing interventions for reactively or proactively aggressive children and adolescents, as earlier shown in numerous studies (see for a meta-analysis, [5]). Since aggression can reflect adverse early experiences in various and complex forms, research on any significant differences in characterizing proactive and reactive aggressive can help to understand the heterogeneity in children’s and adolescents’ aggressive behavior, in particular, person-oriented research based on LPA, such as the present study, because LPA can reveal differences and interactions between multiple indicators that variable-oriented research may mask.

## Figures and Tables

**Table 1:** Goodness-of-fit statistics of the CFA models.

	*χ* ^2^	Scaling	*df*	*CFI*	*TLI*	*RMSEA*	*90% CI*
T1 (February 2014)							
1-factor CFA model	983.117	1.6559	103	0.823	0.793	0.076	[0.072; 0.081]
2-factor CFA model	534.806	1.6328	102	0.913	0.897	0.054	[0.049; 0.058]
4-factor CFA model	273.106	1.5982	97	0.965	0.956	0.035	[0.030; 0.045]
T2 (July 2014)							
1-factor CFA model	288.403	1.5455	103	0.805	0.773	0.093	[0.080; 0.106]
2-factor CFA model	179.064	1.5292	102	0.919	0.905	0.060	[0.045; 0.075]
4-factor CFA model	142.489	1.5111	97	0.951	0.941	0.047	[0.029; 0.064]

*Note*. CFA = confirmatory factor analytic model; χ^2^ = Chi square; df = Degrees of freedom; CFI = Comparative fit index; TLI = Tucker–Lewis index; RMSEA = Root mean square error of approximation; CI = RMSEA 90% confidence interval.

**Table 2:** Results of the LPA models under investigation.

k	#fp	LL	Scaling	AIC	BIC	SABIC	*p*VMRT	*p*BLRT	Entropy
LPA models (T1: February 2014)									
1	8	−8324.331	1.5066	16,664.661	16,706.989	16,681.576	−	−	−
2	13	−7358.297	1.9404	14,742.593	14,811.376	14,770.079	<0.001	<0.001	0.952
3	18	−7063.835	2.1928	14,163.670	14,258.908	14,201.727	0.0788	<0.001	0.955
4	23	−6858.544	2.2514	13,763.088	13,884.780	13,811.716	0.1849	<0.001	0.959
5	28	−6691.085	1.6743	13,438.171	13,586.318	13,497.371	<0.001	<0.001	0.948
6	33	−6576.102	1.8664	13,218.205	13,392.807	13,287.976	0.1261	<0.001	0.945
LPA models (T2: July 2014)									
1	8	−1178.552	1.6376	2373.104	2399.804	2374.457	−	−	−
2	13	−1015.325	2.2339	2056.649	2100.037	2058.847	0.0534	<0.001	0.955
3	18	−952.460	1.8688	1940.920	2000.996	1943.963	0.1686	<0.001	0.981
4	23	−916.706	3.4383	1879.412	1956.176	1883.301	0.8360	<0.001	0.962
5	28	−888.652	1.2717	1833.304	1926.755	1838.038	<0.001	<0.001	0.970
6	33	−870.384	1.4935	1806.769	1916.908	1812.348	0.6710	<0.001	0.911
LTA models									
LTA model with non-invariance	40	−7978.180	1.7919	16,036.360	16,247.999	16,120.931	−	−	0.957
LTA model with invariance	28	−7982.595	1.9805	16,021.190	16,169.337	16,080.390	−	−	0.958

*Notes*. k = number of profiles, #fp = free parameters, LL = model log likelihood; Scaling = scaling factor associated with MLR loglikelihood estimates; AIC = Akaike information criterion; BIC = Bayesian information criterion; SABIC = sample adjusted BIC; *p*VMRT = *p*-value of the Vuong–Lo–Mendell–Rubin likelihood ratio test. *p*BLRT = *p*-value of the bootstrap likelihood ratio test. *p* < 0.001, *p* < 0.01, *p* < 0.05.

**Table 3:** Standardized mean levels of the four aggression factors in the three latent profiles.

**T1 (February 2014)**	**Profile 1** **(4.23%, *n* = 63)**	**Profile 2** **(15.41%, *n* = 226)**	**Profile 3** **(80.36%, *n* = 1179)**
Anger-aggression	1.890	0.935	−0.264
Defensive aggression attribution	1.892	0.860	−0.250
Resource acquisition	3.540	1.274	−0.405
Exercise of power/dominance	2.288	1.007	−0.296
**T2 (July 2014)**	**Profile 1** **(3.85%, *n* = 8)**	**Profile 2** **(14.90%, *n* = 31)**	**Profile 3** **(81.25%, *n* = 169)**
Anger-aggression	1.592	0.767	−0.234
Defensive aggression attribution	1.593	0.868	−0.254
Resource acquisition	3.332	1.205	−0.412
Exercise of power/dominance	1.809	0.906	−0.273

*Notes*. Profile 1: Severe Reactively and Proactively Aggressive (S-RA-PA); Profile 2: Highly Reactively and Proactively Aggressive (H-RA-PA); Profile 3: Moderately Low Reactively and Proactively Aggressive (M-RA-PA).

**Table 4:** Transitions probabilities to time 2 profiles in the LTA of this study.

	Profile 1 (T2)	Profile 2 (T2)	Profile 3 (T2)
Profile 1: S-RA-PA (T1)	0.453	0.547	0.000
Profile 2: H-RA-PA (T1)	0.041	0.514	0.445
Profile 3: M-RA-PA (T1)	0.006	0.066	0.928

*Notes*. S-RA-PA = “Severe Reactively and Proactively Aggressive” profile, H-RA-PA= “Highly Reactively and Proactively Aggressive” profile, M-RA-PA = “Moderately Low Reactively and Proactively Aggressive” profile.

**Table 5:** Sex and age differences in the three aggression profiles under investigation.

	P1 vs. P2		P1 vs. P3		P2 vs. P3	
	Coeff.	*SE*	Coeff.	*SE*	Coeff.	*SE*
T1 (February 2014)						
Sex	−0.587	0.406	−1.834 ***	0.368	−1.247 ***	0.179
Age	−0.040	0.066	0.005	0.059	0.045	0.034
T2 (July 2014)						
Sex	−0.772	0.909	−1.241	0.840	−0.469	0.409
Age	0.001	0.117	0.060	0.096	0.059	0.085

*Notes*. *SE* = standard error of the coefficient; P: Profile; Profile 1: Severe Reactively and Proactively Aggressive (S-RA-PA); Profile 2: Highly Reactively and Proactively Aggressive (H-RA-PA); Profile 3: Moderately Low Reactively and Proactively Aggressive (M-RA-PA). *** *p* < 0.001. Sex: 1 = Boys, 2 = Girls.

## Data Availability

The data presented in this study are available on request from the corresponding author.

## Supplementary Materials

The following supporting information can be downloaded at: https://www.mdpi.com/article/10.3390/children9111733/s1, Table S1: Items and scales of the “Differential Aggression Questionnaire” (in German: Differentieller Aggressionsfragebogen”, DAF; Petermann & Beckers, 2014).
