# Supplementary material for: Reactive and Proactive Aggression among Children and Adolescents: A Latent Profile Analysis and Latent Transition Analysis
Source: Children (Basel). 2022 Nov 11;9(11):1733. doi: 10.3390/children9111733 (PMC9688458; doi:10.3390/children9111733)
Supplement: Supplementary file 1 [file children-09-01733-s001.zip › children-1961368-supplementary.pdf]

Online Supplements

Table S1.
Items and scales of the "Differential Aggression Questionnaire" (in German: Differentieller Aggressionsfragebogen", DAF; Petermann & Beckers, 2014)

English translation	German original item content
**Anger-aggression**	
1. How often have you become very angry when someone called you names?	1. Wie oft bist du sehr wütend geworden, wenn dich jemand beschimpft hat?
2. How often have you thrown a tantrum?	2. Wie oft hast du einen Wutanfall bekommen?
3. How often have you broken something because you were so angry?	3. Wie oft hast du etwas kaputt gemacht, weil du so wütend warst?
4. How often have you yelled at someone when this person teased you?	4. Wie oft hast du jemanden angeschrien, wenn dich diese Person geärgert hat?
**Defensive aggression attribution**	
1. How often have you been involved in a quarrel for which you could do nothing?	1. Wie oft bist du in einen Streit verwickelt worden, für den du nichts konntest?
2. How often have you hit someone just to defend yourself?	2. Wie oft hast du jemanden nur deshalb geschlagen, um dich zu verteidigen?
3. How often have you beaten when someone else started?	3. Wie oft hast du dich geprügelt, wobei jemand anderes angefangen hat?
4. How often have you fought even though you didn't want to?	4. Wie oft hast du dich geprügelt, obwohl du nicht wolltest?
**Resource acquisition**	
1. How often have you threatened someone to do what you want?	1. Wie oft hast du jemandem gedroht, damit diese Person macht, was du willst?
2. How often have you harmed someone to gain an advantage?	2. Wie oft hast du jemandem geschadet, um einen Vorteil zu erhalten?
3. How often have you threatened someone to get what you want?	3. Wie oft hast du jemanden bedroht, um zu erreichen, was du willst?
4. How often have you intimidated someone into doing what you want?	4. Wie oft hast du jemanden eingeschüchtert, damit diese Person macht, was du willst?
**Exercise of power/ dominance**	
1. How often have you asked others to tease someone?	1. Wie oft hast du andere aufgefordert, jemanden zu ärgern?
2. How often have you called someone names just for fun?	2. Wie oft hast du nur zum Spaß jemanden beschimpft?
3. How often have you asked others to harm someone?	3. Wie oft hast du andere aufgefordert, jemandem etwas anzutun?
4. How often have you hit someone just for fun?	4. Wie oft hast du nur zum Spaß jemanden geschlagen?

Note. All items responded to on a 4-point response scale ranging from "never" (0), "rarely" (1), "more often" (2) to "often" (3).
